# Implementing Systemic Innovation Strategies for a More Sustainable Future: The Case of Three Overseas Countries and Territories

**DOI:** 10.3389/frma.2021.801789

**Published:** 2022-01-24

**Authors:** Renato Toffanin, Milan Jezic von Gesseneck

**Affiliations:** ^1^Advanced Research Centre for Health, Environment and Space (ARCHES), Castellana Grotte, Italy; ^2^Chamber of Commerce Belgium Luxembourg South East in Europe, Brussels, Belgium

**Keywords:** systemic innovation, innovation strategies, innovation policies, backcasting, policy learning, overseas countries and territories (OCTs)

## Abstract

Addressing sustainability issues requires a radical systemic change across multiple dimensions, including policy, culture, and civil society. This also implies that no blueprints for governing critical sustainability issues both at the local and global levels exist. As a result, rather than imposing decisions, policymakers should engage in a learning process. In this paper, we contend that appropriate policies should be developed and fine-tuned over time through a collective, social endeavour. To support this hypothesis, the study focuses on a shared methodology based on backcasting, a specific type of foresight, to facilitate policy learning (and thus policymaking) within a wide range of territories, regardless of their wealth, geographic characteristics and internal political organisation. This methodology was developed over a three-year period as part of the Territorial Strategies for Innovation (TSI) programme. The overall objective of our assignment was to build capacity and raise awareness within the EU's Overseas Countries and Territories about policymaking and implementation of innovative approaches to development. This innovative approach, which incorporates a systemic innovation perspective, highlights new options and opportunities for adopting and implementing adequate policies to positively impact sustainable development and long-term transformative change. Using empirical examples from Anguilla, Curaçao, and New Caledonia, the paper focuses on the learning processes required to deal with complexity and uncertainty in these remote territories. We conclude by discussing the potential implications of this foresight approach for the sustainable development and transformation of other less-favoured regions and territories.

## Introduction

Sustainable development is often seen as a process within the context of globalisation, encompassing the economic, social, and environmental dimensions (Beumer et al., 2018). In recent years, the rapid pace of globalisation has accelerated change in all of these dimensions thus causing trends and processes in various spheres to interact with one another in unpredictable ways. Particularly complicated problems, however, make it increasingly difficult to address individual issues without facing the risk of unintended consequences (Stiglitz, 2002; Scholte, 2005). This uncertainty presents a further challenge for decision makers tasked with formulating sustainable policies that effectively address intertwined problems (Martens and Raza, 2010). Indeed, this also raises the question of a more inclusive and holistic approach to innovation policies that takes up such a complex issue (OECD, 2015).

The spatial implications of globalisation and rapid pace of innovation pose a formidable challenge for places not favourably situated to benefit from new forms of knowledge-intensive growth such as peripheral regions and territories (Isaksen and Karlsen, 2016; Rodriguez-Pose and Wilkie, 2017). In fact, these remote areas generally lack the preconditions for innovation typically associated with urbanisation economies (Tödtling and Trippl, 2005; Isaksen and Trippl, 2017). As a consequence, suitable forward-looking and adaptive policies should be adopted to foster sustainable development and transformation in ultra-peripheral territories, such as the European Union (EU) Overseas Countries and Territories (OCTs).

Associated with the European Union, the OCTs comprise 13 islands, which have special constitutional relationships with Denmark, France and the Netherlands. Until 31 January 2020, there were additional 12 islands linked to the United Kingdom^1^. Spreading from the poles to the tropics, they play an important role as outposts of the European Union in the areas where they are located, but do not, however, form part neither of the EU territory nor of the EU single market. As islands, they face common systemic issues such as loss of population, geographical isolation, high transport costs, heavy dependency on imports, and limited economic activity, often focussed on few economic sectors (e.g., tourism and fisheries). One major issue is the population decline caused by a lack of job opportunities, particularly for young and talented people. Climate change and energy dependence are two other pressing issues confronting the OCTs. To compensate for these disadvantages, they frequently benefit from remarkable natural assets such as sea resources, unique biodiversity, the potential for renewable energy production, and an appealing climate for tourism. Furthermore, these remote islands are more reliant on their own resources, with greater involvement of the local communities.

The OCTs, which are located in the Caribbean Sea as well as the Indian, Pacific, and Atlantic oceans, have a distinct political and institutional status. They differ, in fact, from the EU outermost regions (ORs), which are politically and economically integrated into a parent-state, and from the small island developing states (SIDS), which are fully-fledged independent countries. Like this latter group of islands, the OCTs must address critical sustainability issues. This entails putting their economy on a sustainable development path as well as increasing competitiveness, reducing economic vulnerability to natural and external shocks, increasing environmental resilience, improving cooperation with neighbouring islands and, where possible, better integrating their economy into the regional and global economies.

In a recent publication, we have emphasised the importance of a systemic approach to innovation for the sustainable development of the OCTs (Jezic von Gesseneck et al., 2018). Specifically, innovation system foresight (Andersen and Andersen, 2014), in conjunction with backcasting (Robinson, 1982; Dreborg, 1996), has been proposed to assist the governments of selected OCTs (as well as the other OCTs) in defining their innovation strategies. The difficult challenge now refers to the design and implementation of specific innovation policies in order to avoid distortions and government failures. To better harness local potential innovations, the overall aim of this paper is to explore options and opportunities for innovation policymaking to positively impact the sustainable development and transformation of these emerging economies (Kuhlmann and Ordóñez-Matamoros, 2017). Given the complexity of the issues, special attention is paid to the learning processes required to achieve these ambitious policy objectives (van Mierlo et al., 2020). Therefore, the broader research question addressed by this study is as follows:

- How can learning help facilitate and trigger sustainable development and transformation in individual OCTs?

Our research design is a multiple-case inductive study. Using documentation, direct observations and secondary data sources, we present empirical evidence from three selected OCTs, namely Anguilla, Curaçao, and New Caledonia. This paper is divided into five sections. Section Conceptual Framework and Background follows the introduction and is devoted to the background literature and conceptual framework. We set out the conceptual framework linking systemic innovation and policymaking to capacity building and policy learning. In section Methodological Approach, we address the methodologies while in section Findings, a synthesis of the case study results and the lessons learned are presented. Section Discussion and Conclusions discusses the findings and concludes the paper with potential implications of the proposed foresight approach for improving the innovation performance of Europe's less-favoured regions.

## Conceptual Framework and Background

The EU OCTs like other countries and territories around the world are pursuing innovation-led growth and sustainability while grappling with systemic issues and pressing challenges. Such ambitious objectives undoubtedly necessitate appropriate and effective innovation policy interventions (Mowery et al., 2010; Steward, 2012; Weber and Rohracher, 2012; Hekkert et al., 2020; Larrue, 2021). They also necessitate a significant transformation in the modes of innovation themselves (Leach et al., 2012; Stilgoe et al., 2013) as well as an appropriate framework for addressing the innovation-related challenges (Weber and Truffer, 2017). As a result, it is clear that these objectives can only be met through a systemic approach that includes all of the critical elements and functions of the emerging OCT innovation systems. In this study, we adopt the notions of socio-technical and innovation systems defined by Borrás and Edler (2014a, p. 11) as “*articulated ensembles of social and technical elements which interact with each other in distinct ways, are distinguishable from their environment, have developed specific forms of collective knowledge production, knowledge utilization and innovation, and which are oriented towards specific purposes in society and economy.”*

### Innovation Policymaking

In their analysis on the rise of systemic instruments, Smits and Kuhlmann (2004) identified three major trends that characterise the changing nature of innovation processes and systems: (i) the linear model's demise, (ii) the rise of the systems approach, and (iii) the inherent uncertainty and need for learning. They also claimed that systemic instruments are increasingly being used to supplement traditional approaches to innovation policy. The traditional rationale for innovation policy, however, has recently changed. Innovation policy is expected to more explicitly contribute to addressing societal demands (Boon and Edler, 2018) and even to responding to grand challenges^2^ (Kuhlmann and Rip, 2014, 2018). As a result, challenge-oriented policymaking necessitates broader, more open and flexible approaches (Daimer et al., 2012; Kallerud et al., 2013).

To make innovation policy more effective, policymakers may need to combine various instruments in an appropriate innovation policy mix (Cunningham et al., 2016; Kivimaa and Kern, 2016; Rogge and Reichardt, 2016). This entails shifting the policy mix away from direct R&D funding towards policy instruments addressing major challenges (Edler and Fagerberg, 2017; Fagerberg, 2017). This may necessitate a greater emphasis on emerging policy instruments such as public-private partnerships (OECD, 2008; Wieczorek and Hekkert, 2012; Kristensen and Scherrer, 2016), public procurement (Edler and Georghiou, 2007; Edquist and Zabala-Iturriagagoitia, 2012; Edquist et al., 2015) and foresight (Cagnin et al., 2012; Frau, 2020). As Edler and Fagerberg (2017) point out, selecting the right policy instruments necessitates a thorough understanding of the systemic bottlenecks that impede the generation and diffusion of innovations, such as insufficient skills/competences, a lack of interaction or uncertainty about future demand. Furthermore, developing a more holistic innovation policy necessitates a knowledge of the nature and dynamics of innovation processes in the innovation systems (Borrás and Edquist, 2019). It is obvious that innovation policies aimed at transforming an innovation system towards sustainability necessitate enhanced capabilities of policymakers and other relevant actors involved in innovation policymaking.

As Mazzucato (2018) points out, systemic challenge-driven policies must be founded on a sound and clear diagnosis and prognosis (foresight). This necessitates not only the identification of missing links, failures and bottlenecks—the weaknesses or challenges of the concerned innovation system—but also recognition of the system's strengths. In order to explore future opportunities, understand how strengths can be used to mitigate systemic weaknesses, and set goals, foresight is essential. A few years ago, the concept of innovation system foresight has been introduced by Andersen and Andersen (2014). By including a systemic and evolutionary understanding of innovation, this foresight approach appears well-suited to guide the transformation of innovation systems towards desirable directions (Andersen and Andersen, 2017). In a previous study (Jezic von Gesseneck et al., 2018), we used a combination of innovation system foresight and backcasting (Robinson, 1982; Dreborg, 1996) to assist OCT governments in developing innovation strategies and policies to address local and global challenges. Indeed, the current orientation to contemporary challenges, as expressed for example in the Sustainable Development Goals (United Nations, 2015), is a prerequisite for more appropriate innovation policies (Schot and Steinmueller, 2018; Diercks et al., 2019). Challenge orientation implies that innovation policy requires direction (Lindner et al., 2016) and normative decisions (Daimer et al., 2012; Schlaile et al., 2017). It also implies that a broader range of actors is involved in the formulation and implementation of policy measures. Plausibly, multiple heterogeneous actors have diverging and conflicting visions, interests, norms, and expectations regarding various sustainability goals (Shove and Walker, 2007; Meadowcroft, 2009; Smith and Stirling, 2010). Hence, addressing today's social and environmental challenges necessitates a radical systemic change across multiple dimensions, including policy, culture and civil society (Geels, 2012). This implies that, as pointed out by Korten (1980) many years ago, there are no blueprint approaches to the governance of critical sustainability issues. As a result, rather than imposing final solutions, policymakers should engage in a learning process.

### Policy Learning

Policy learning studies are based on the broad definition of learning as “*the updating of beliefs based on lived or witnessed experiences, analysis or social interaction”* (Dunlop and Radaelli, 2013, p. 599). More specifically, Borrás (2011) argues that policy learning refers to the specific process in which knowledge is used in the concrete development of policy formulation and implementation. Policy learning, according to Biegelbauer (2013), can be defined as a change of policy-relevant knowledge, skills, or attitudes, which are the result of new information or the assessment of past, present, or potential future policies. Based on the previous work by Bennett and Howlett (1992); Borrás (2011) explores the links between learning and organisational capacity. In her work on innovation policies, she identifies three levels of policy learning and argues that their effects on innovation systems are related to the organisational capacity of the relevant actors to implement change. The first level of learning is government learning or the learning that government and public organisations in the innovation system adopt with respect to organisational practises. This level relates to the administrative capacity of the government itself and mainly concerns the ability to develop, direct, and control its human resources to support the discharge of public policy and programme responsibilities (Donahue et al., 2000). The second level of learning is policy network learning, which corresponds to the learning processes of major stakeholders and governmental actors regarding the interaction between policy and the innovation system. This level, which is primarily intended to identify system failures, necessitates analytical capacity. According to Howlett (2009), analytical capacity describes the ability of an organisation to produce valuable policy-relevant research and analysis on topics of their choosing. The third level of learning is social learning (Hall, 1993), which involves a diverse group of socio-economic actors within the innovation system. This type of learning focuses on avoiding potential governance failures within the innovation system. In this case, the required capacity is more diffused than in the previous two examples because it necessitates a certain degree of reflexivity in a widely dispersed set of policy actors. As Dunlop and Radaelli (2018a, p. 598) point out, reflexive capacity is being developed “*where issues are complex but knowledge is contested, expertise is disparate and spread across society. In these instances, capacity challenges are met through wide-ranging social interactions and collective puzzling. The key learning mechanism is triggered by a reconsideration of one's beliefs and preferences.”*

### Capacity Building

Policy learning and change clearly depend on the level of organisational capacity of key policy actors. It follows that organisational capacity is inextricably linked to the central concern of governance, namely the ability to make policy decisions (Peters and Pierre, 2007). As a result, when we consider organisational capacity we are concerned with the resources and competences deployed to develop and adjust the policy instruments and procedures necessary to achieve policy goals. Hence, policy learning can be viewed as an important component of the ongoing process of capacity building aimed at achieving longer-term sustainability goals in individual OCT contexts. Thus, our research efforts heavily focus on competence and competence building from the standpoint of systemic innovation, and particularly from the angle of innovation policy. Indeed, there is widespread agreement that competences play a critical role in the dynamics of innovation systems and in the processes of governing change towards more sustainable directions (Geels et al., 2008). For this reason, policy learning is critical for acquiring and developing the necessary competences, intended here as the set of knowledge, skills and expertise that policy actors are expected to achieve in order to foster sustainable transformation. Thus, through the lens of policy learning, we investigate how to help the individual OCTs overcome organisational and institutional barriers, and establish a common orientation for innovation policies. Developing and implementing these policies require a deep understanding of the nature and dynamics of innovation processes as well as a thorough understanding of the problems that the various OCTs face. As a result, special emphasis is placed on identifying concrete policy issues, as well as selecting specific systemic instruments and incorporating them into effective mixes^3^.

### Backcasting

Preparing for the future involves foresight, i.e., the disciplined exploration of alternative futures. With regard to policymaking, foresight represents a prominent systemic instrument (Daimer et al., 2012) and the benefits of this participatory approach for the learning capacity of innovation systems have been emphasised by various authors (Da Costa et al., 2008; Cagnin et al., 2012; Andersen and Andersen, 2014; Rosa et al., 2021). In fact, foresight engages diverse actors in a joint learning process, resulting in future-oriented attitudes and linkages and, ultimately, improving the responsiveness of the innovation system towards future challenges and options. Furthermore, by facilitating the participation of civil society in innovation governance, it can improve the transparency and legitimacy of the policymaking process, as well as the acceptance and credibility of policy decisions (Barré, 2001).

Foresight activities that effectively intend to address fundamental challenges need to consider discordant voices and multiple beliefs. Among the various forms of foresight, backcasting^4^ is particularly well-suited to facilitating the participation of multiple heterogeneous actors (Quist, 2007). Furthermore, because it is oriented towards alternative futures and policy goals rather than likelihood, backcasting is explicitly normative (Robinson, 1982; Dreborg, 1996; Börjeson et al., 2006). For this reason, it is well-suited to addressing sustainability issues with stakeholder participation (Carlsson-Kanyama et al., 2008; Höjer et al., 2011; see also Vergragt and Quist, 2011). This approach also assumes that both vision development and pathway development encompass social learning processes (Robinson, 2003). From a policy analysis perspective, this learning process implies second order within the policy community (Bennett and Howlett, 1992; May, 1992; see also Goyal and Howlett, 2018). Thus, for social learning to be sustained, backcasting analysis must be highly integrative and able to unveil some of the higher-order consequences and trade-offs associated with various choices. This means that in second-order learning the participants can not only change their understandings of a specific policy option but also their beliefs about the nature of the problem being addressed (Argyris and Schön, 1978; van de Kerkhof, 2006). As a result, rather than attempting to reach a consensus, the dialogue process should facilitate the exploration of various solutions to some identified problems. Furthermore, the inclusion of multiple actors in backcasting studies can aid in the translation of backcasting outcomes into the actual implementation of policy measures by the key agents tasked with promoting change (Wang, 2011). Based on the foregoing, we propose backcasting as a key strategic tool for improving the policy analytical capacity (Howlett, 2009) of the concerned OCT governments. As a consequence, the following more specific research questions are posed and addressed:

- How can backcasting aid in the implementation of more effective innovation policies in these less-favoured territories?
- How can backcasting help align the policy mixes to their evolving innovation strategies?

The next sections are primarily devoted to these questions. With a focus on three different OCT contexts, we describe the approach used to assist the respective OCT governments in acquiring new knowledge aimed at sustainable development and systemic transformative change. We also consider a specific governance arrangement that could be implemented to steer innovation in the right direction.

## Methodological Approach

This innovative approach was developed over a 3-year period as part of the Territorial Strategies for Innovation (STI) programme, which was funded by the 10th European Development Fund (EDF) and ran from April 2014 to April 2020. The overall programme objective was to build capacity and raise awareness within the EU's Overseas Countries and Territories about policymaking and implementation of innovative approaches to development. To that end, it was critical to strengthen the policy capacity (Peters, 1996; Parsons, 2004; Wu et al., 2015) of OCT governments facing local level systemic challenges as well as global challenges such as climate change, energy security and social justice. To improve the implementation and impact of the STI programme, special attention was paid to the development of specific actions through the dynamic participatory process outlined below.

### Foresight Methodology

**Figure 1:**
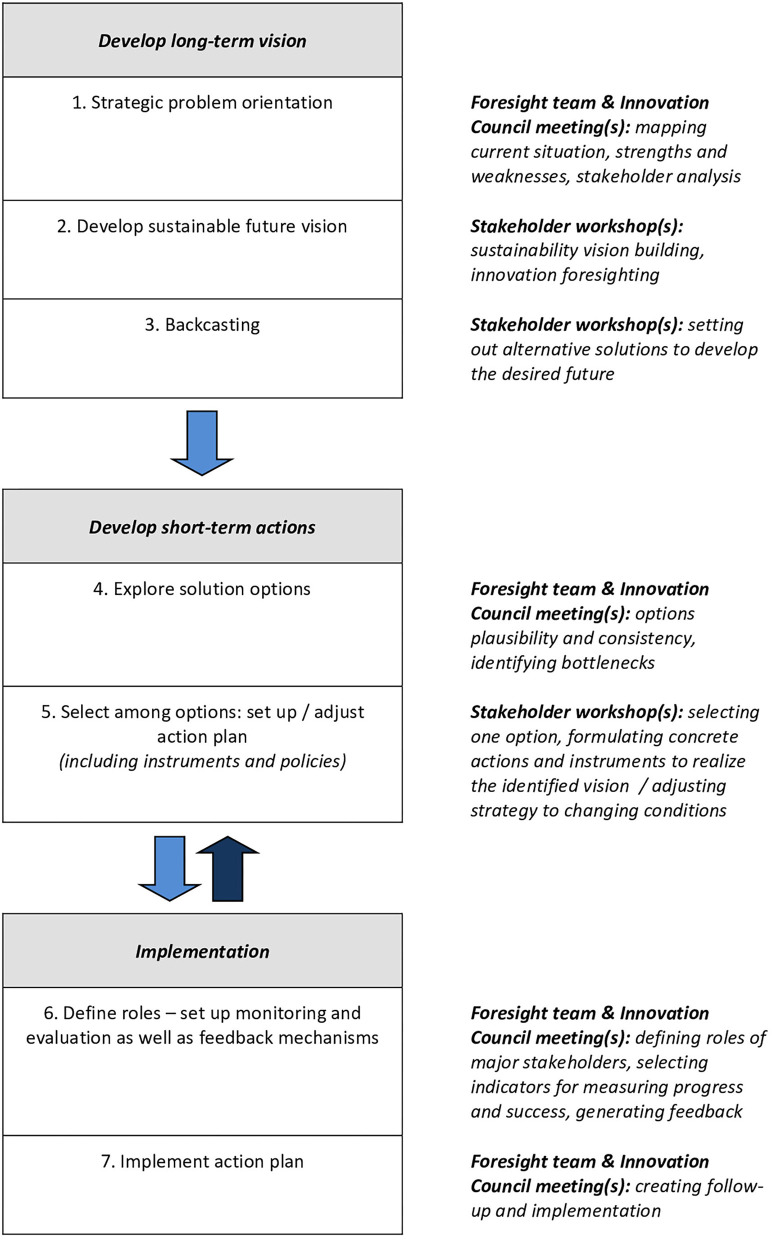
Key steps of the proposed foresight methodology, which also includes a feedback loop to shape innovation policy mixes over time; adapted from Jezic von Gesseneck et al. (2018).

The entire process, which was based on the concept of innovation system foresight (Andersen and Andersen, 2014), was divided into three phases, namely, pre-foresight (planning phase), foresight (main phase), and post-foresight (implementation phase). Each had a number of steps. For all OCTs, the intended time horizon was 15 years or more that is 2030 and beyond, with developing both the short-term and long-term actions being a key component of the entire foresight study. During the foresight phase, specific activities were carried out to gain a comprehensive understanding of the OCT specificities and to assess the interactions between global trends and local challenges. The applied foresight methodology combined different methods such as literature review, SWOT and PESTLE analyses and backcasting. Institutional and non-institutional stakeholders were involved from the start of the foresight exercise and multiple public-private consultation workshops were held on several occasions in Brussels and the respective OCTs. Following these events, there were online interactions. Figure 1 depicts a schematic representation of the seven-step foresight approach^5^ used in the study. Steps 1–3 were designed for developing a long-term strategic vision. In particular, a deep analysis of the current situation (Step 1) was performed by the foresight team of the TSI programme (3 persons) in collaboration with the Innovation Council of the concerned OCTs. A stakeholder analysis was also carried out, which included stakeholders from both the demand and supply sides. Selected stakeholders participated in the first series of stakeholder workshops (Step 2), which aimed to identify sustainable paths for future innovation development. The number of participants ranged from 32 to 146. During these day-long workshops, a diverse range of actors from the public and private sectors were encouraged to think creatively about long-term solutions to major challenges. The outcomes were used for vision building. These visions were assessed based on economic growth and environmental sustainability. All stakeholders were invited to a second round of sectoral and cross-sectoral workshops (Step 3) where visions and assessment results were discussed for achieving the desired future state. Backcasting techniques were used in this series of workshops designed to promote reflexive learning. Steps 4 and 5 were intended to clarify the short-term actions required to achieve that future state. The various solutions were further defined and analysed (Step 4) by the foresight team in collaboration with the Innovation Council of the respective OCTs. A third round of stakeholder workshops (Step 5) was held to determine the preferred option and formulate concrete actions and policy recommendations for designing and adjusting the innovation strategy. Steps 6 and 7 (corresponding to the post-foresight phase) deal with the implementation of the innovation strategies in the various OCTs. Step 6 of the initial foresight methodology was improved by the addition of a feedback mechanism, as proposed by Edmondson et al. (2019), to provide policy-mix directionality in line with the evolving systemic innovation strategy.

**Figure 2:**
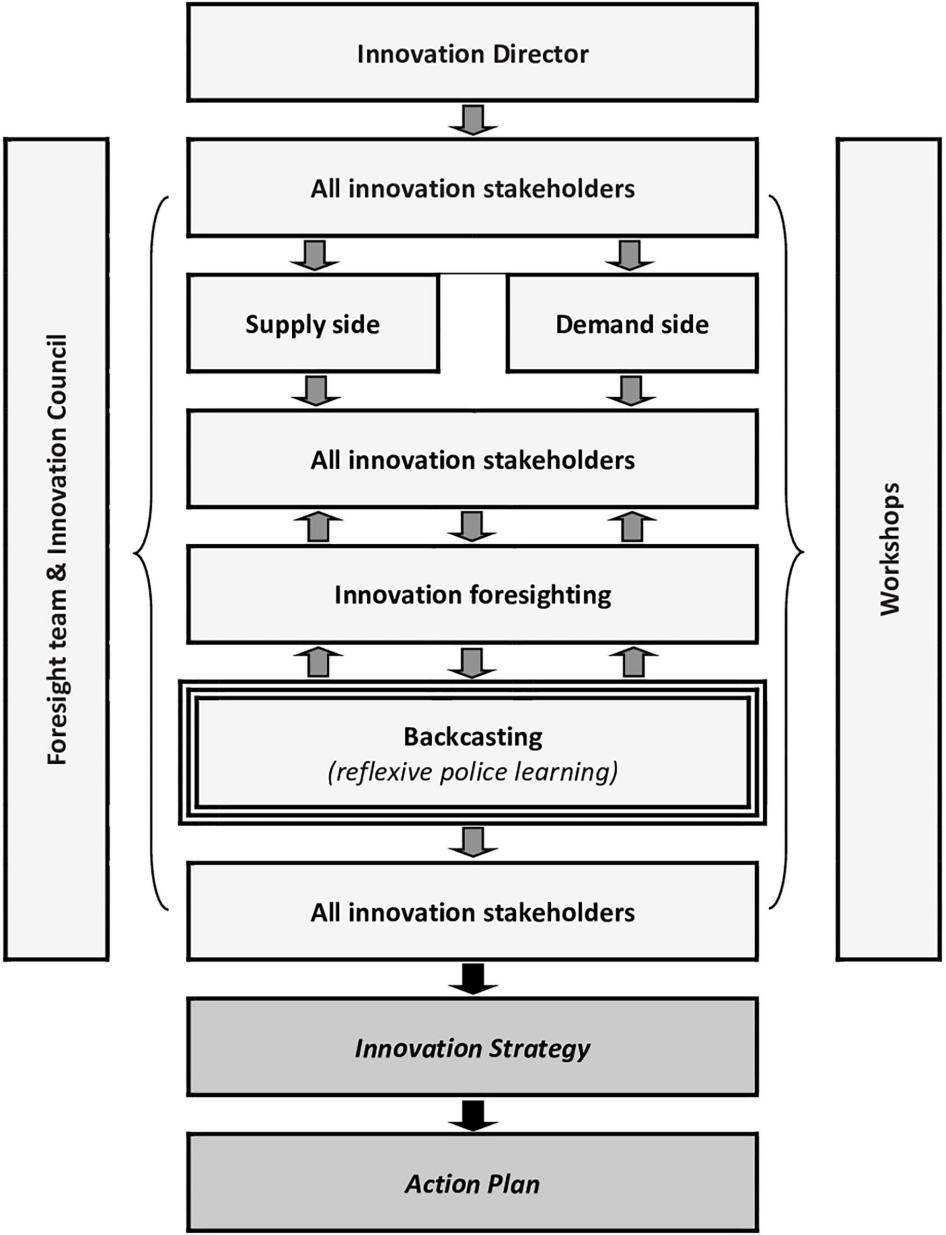
Decision-making process with the participation of multiple stakeholder groups. Backcasting is crucial for inducing reflexive learning in the respective policy communities; adapted from Jezic von Gesseneck et al. (2018).

Figure 2 depicts the stakeholder involvement and the decision-making process used for Curaçao and New Caledonia, which included up to 10 stakeholder workshops. A simplified participatory mechanism with fewer workshops was used for Anguilla.

### Case Study Methodology

To get a complete picture of the overall OCT situation, we adopted the case study methodology (Yin, 2014) to better understand the differences and similarities between the various OCT contexts. We used an inductive, multiple-case design because multiple cases provide a wider base of knowledge and allow for a more in-depth exploration of research questions (Eisenhardt and Graebner, 2007). The study's empirical focus is on three OCTs: Anguilla, Curaçao, and New Caledonia. These OCTs were selected for two primary reasons. Firstly, they are among the OCTs that are making the most progress in developing innovation policy action plans. Secondly, these OCTs exhibit a variety of social, economic, institutional, cultural, and historical characteristics that make them interesting for comparison. We compared these rather different cases by focusing on their capacity to learn, innovate, and adapt to local and global challenges. This case study is based in part on the participant observations of one of the authors. Participant observation is a qualitative method for collecting data, widely used by social scientists (Spradley, 1980; DeWalt and DeWalt, 2010). The data analysis process was highly iterative and used conventional approaches for inductive research. We began with a careful analysis of each case focusing on the specific context in which innovation should take place. We initially analysed the cases independently one from the other. Then we turned to cross-case analysis to compare the insights from each case and see patterns on a larger scale.

## Findings

Envisioning a common sustainable future (i.e., 2030 and beyond), the investigated OCTs are striving towards long-term sustainability outcomes while exploring multiple and diverse policy pathways in the short term. Their main point of commonality is a high level of vulnerability to external economic shocks and environmental impacts. In this section, we present the main research findings for the concerned OCT, emphasising the key characteristics and learning processes involved in their transition towards sustainable development and transformation. Finally, we provide an overview of the lessons learned from implementing our systemic approach to innovation.

### Anguilla

Anguilla is a British overseas territory in the Eastern Caribbean. Anguilla has few natural resources and the economy of this tiny island is based primarily on luxury tourism, offshore banking, lobster fishing, and remittances from emigrants. The local authorities have made significant efforts to expand the offshore financial sector. Because of its reliance on tourism, Anguilla's economy is extremely vulnerable to external shocks, as evidenced by the 5-year period of contraction following the global financial crisis. In 2013, the economy began a slow but steady recovery. Limited economic growth and over-dependence on luxury tourism emphasise the need to diversify economic activity. There is a high level of education and entrepreneurial spirit on the island. However, the majority of high achievers go on to further their education in North America or the United Kingdom, and they frequently do not return. The government is concerned with facilitating the economic empowerment of Anguilla's youth and creating an environment conducive to entrepreneurship and innovation. Damaging storms are a major driver of change for this remote territory. On 6 September 2017, Anguilla was hit by Irma, a Category 5 hurricane. The impact was immediate and devastating on an island that is heavily reliant on travel and tourism—it contributed 61.6 per cent of Anguilla's GDP in 2017. The island was then hit by the COVID-19 pandemic. While still recovering from this second devastating event, Anguilla is now implementing its systemic innovation strategy, which aims to accelerate the pace of socio-economic and sustainable growth in particular. Tourism, fisheries, agriculture, and STEM education are top priorities. The appointment of an innovation director and the formation of an innovation advisory board were critical steps in the definition of the innovation governance at the start of the TSI programme. This advisory body included members of the civil service of the government, the chamber of commerce and industry, private business, and other key local stakeholders. Their expert advice has proven to be extremely beneficial in terms of providing instrumental knowledge to guide policymakers towards specific solutions, particularly in the area of entrepreneurship. Backcasting activities, on the other hand, have been fundamental in including a broader range of actors in the decision-making process. In particular, in a reflexive setting, institutional, and non-institutional stakeholders (a total of 32 participants) were able to define the strategic priorities of the innovation policy and outline the enabling starting conditions for the transformation of the Anguillian innovation system. The identification of problems and policy instruments (Table 1) was the main initial outcome of this collective learning endeavour. Social learning proved to be extremely helpful in shaping a specific policy mix capable of initiating a series of coordinated short-term actions aimed to overcome major systemic bottlenecks. The chosen core instruments were increased investments in human capital, which were complemented by other instruments to promote an entrepreneurial and innovation culture among the local youth and female communities. Three pilot projects, in particular, were launched and successfully implemented on the basis of a shared vision of an innovative and sustainable island resilient to climate change. The specific goals of this preliminary experimentation included the economic empowerment of the local youth community through dedicated training programmes as well as the alignment of tourism with the innovation priorities of other critical sectors such as agriculture, fisheries, and cultural heritage.

**Table 1:** Main systemic innovation problems in the concerned OCTs and relevance of policy instruments to mitigate them.

**Systemic innovation areas**	**Systemic problems**	**Policy instruments**	**Anguilla**	**Curaçao**	**New Caledonia**
Knowledge creation and R&D	No or insufficient private investment in R&D	Innovation vouchers as financial subsidies	●●❍	●●●	●●●
		Fiscal incentives	●●❍	●●❍	●●❍
		Innovation-oriented public-private partnerships (PPPs)	●●●	●●●	●●●
Education, training and skills	Insufficient skills and competences due to low levels of education	Three-pillar blended learning including life-long learning and vocational training	●●●	●●●	●●●
	Dependence on foreign knowledge in science, technology, engineering and mathematics (STEM)	Three-pillar blended education programmes in cooperation with recognised foreign STEM platforms	●●●	●●●	●●●
	Brain drain	Competences building to reverse brain drain through three-pillar blended learning	●●●	●●●	●●●
New product markets and quality requirements (demand-side)	Lack of innovation dynamics in the economy and in the public sector	Innovation-friendly public procurement	●●❍	●●❍	●●❍
Entrepreneurship	Weak levels of entrepreneurship and new entrants in the economy	Promoting entrepreneurial culture	●●●	●●❍	●●❍
		Support to start-ups including access to seed and venture capital	●●●	●●●	●●●
		Dissemination of best practises of innovation management	●●❍	●●❍	●●❍
Innovation networks	Weak co-operation between innovating organisations	Developing local interactions and networks, particularly through PPPs	●●●	●●●	●●●
		Financing network activities to facilitate reflexive governance and social learning (e.g., backcasting)	●●●	●●●	●●●
		Specific instruments to foster the embeddedness of small and medium-sized enterprises (SMEs) in networks	●●●	●●●	●●●
Regulatory frameworks	Inadequate IPR regime	IPR measures	●❍❍	●●❍	●●❍
	Lack of innovation friendly economic regulations	Competition enhancing regulations	●❍❍	●❍❍	●❍❍
	High level of uncertainty	Implementing technical standards	●●❍	●●❍	●●❍

*Adapted from von Borrás and Edquist (2016, 2019); Jezic von Gesseneck et al. (2018)*.

*●●●Major relevance; ●●❍Moderate relevance; ●❍❍Minor relevance to the transformation of the local innovation system towards the desired direction*.

### Curaçao

This Dutch overseas territory is strategically located in the Southern Caribbean between the Americas and being part of the Kingdom of the Netherlands has close ties with the European Union. Most of Curaçao's GDP results from services. Oil refinery, tourism, offshore finance, and transportation and communications are the mainstays of this small island economy. Curaçao has beautiful beaches but few natural resources such as poor soil and insufficient water supplies. Almost all consumer and capital goods are imported, with the majority coming from the United States, the Netherlands and Venezuela. Despite only a slight increase in GDP over the last decade, Curaçao has a high per capita income and a well-developed infrastructure when compared to other Caribbean islands. Curaçao's multi-cultural and multi-lingual society as well as its skilled workforce facilitate business opportunities with the rest of the world. On the other hand, Curaçao's 19.1 per cent unemployment rate (in 2020) is among the highest in the Caribbean region. In this particular socio-economic context, the government is concerned with increasing the level of entrepreneurial activity on the island and creating a more inclusive society. The local authorities are also concerned about the negative impact on health and the environment related to the refinery^6^.

Curaçao is currently implementing its systemic innovation strategy, which aims to reinforce the tourism and ICT sectors in particular. It also aims to sustain emerging sectors such as renewable energy, transnational education and creative industries. The appointment of an innovation manager in 2014, followed by the formation of an innovation advisory board, was a fundamental step in the definition of the innovation governance. The latter initially comprised representatives of the government, the chamber of commerce and industry, the university and other relevant stakeholders such as banks and business angels. The advisory body has expanded over time to include new experts in order to better examine and discuss the technical complexities of specific systemic issues impeding the development of the Curaçaon innovation system. In this specific OCT context, the advisory board has also reinforced the relationship between the government and the local epistemic community^7^ On the other hand, involving multiple actors in the backcasting activities has proven to be extremely beneficial in terms of triggering a reflexive learning process. In this particular case, social learning was crucial in shaping a specific policy mix able to initiate a series of short-term actions targeting competitiveness and sustainability. Table 1 lists the main systemic instruments with the potential to mitigate the identified problems and initially guide the transformation of this Caribbean island towards the desired direction. The selection of specific policy instruments was the result of a complex process involving a large number of local stakeholders (a total of 104 participants) with diverse interests and points of view. In the end, the core instruments chosen were increased investments in Curaçao's technology-driven companies through dedicated programmes to develop local interactions and networks. The inclusion of a systemic instrument to aid in the formation of public-private partnerships^8^ was particularly significant (PPPs). These collaborations enabled the launch of several pilot projects, including the “Start-up Launchpad” programme, which aimed to boost local start-ups by providing training and connecting them with potential investors.

### New Caledonia

This French overseas territory in the South Pacific is a special collectivity. New Caledonia has 11 per cent of the world's nickel reserves, making it the world's second-largest reserves. Only a small portion of the land is suitable for cultivation and food accounts for ~20 per cent of total consumer goods imports. In addition to nickel mining and metallurgy, tourism, services, fisheries, and aquaculture are keys to the health of the economy. Between 1998 and 2006, New Caledonia experienced strong and sustained economic growth. Nowadays, the average household income is quite high. However, this prosperity is largely dependent on nickel mining and subsidies from mainland France. The New Caledonian economy is exposed to the high volatility of nickel prices and suffers from a lack of productivity gains, insufficient competitiveness and strong income disparities. Persistent inequalities among the population are a major source of concern for the Caledonian society. Despite the fact that this OCT has a well-educated workforce, high rates of poverty and illiteracy affect the indigenous community^9^. A major source of concern also derives from the nickel industry, which poses a serious threat to the stunning natural environment of this remote archipelago.

New Caledonia is now putting its systemic innovation strategy into action, with a focus on tourism, the digital economy, agriculture, natural resources, and health. The ambition of the local authorities is to create an innovation-friendly environment to boost the competitiveness of the Caledonian enterprises and promote regional integration. 2015 was a watershed year for innovation in New Caledonia, with the launch of several dedicated initiatives, as well as the explicit desire on the part of the government and the provinces, but also the State, to improve the governance of innovation to truly make it a lever for economic and social development, through specific public policies. These include the government's appointment of an innovation director at the end of 2014 and the formation of an innovation advisory board at the start of 2015, namely the *Comité Consultatif de l'Innovation* (CCI). The CCI brought together representatives of the State, the government, the three provinces, the three chambers of commerce, trade unions, private and public funders, research institutes, and other members of the local epistemic community. The newly formed board played a pivotal role in the management of the backcasting activities, which resulted in a shared understanding of the main problems affecting the Caledonian innovation system. The identification of specific policy instruments was also a major initial outcome of these activities (Table 1), which involved policy actors, representatives of the public administration and a large number of non-institutional stakeholders in an exemplary learning process. Social learning was crucial in developing a specific policy mix capable of initiating a series of short-term actions in the education and research sector, which is a cornerstone of this remote territory. Instrument selection was, in fact, a rather complex activity that required the engagement of a large number of stakeholders (a total of 146 participants) with different beliefs and preferences. In the end, the core instruments chosen were increased investments in human capital through dedicated training programmes to ensure a proper match between skills and jobs. In this case, as well, the inclusion of a systemic instrument to aid in the formation of public-private partnerships was extremely beneficial. PPPs facilitated the launch of a number of innovation-oriented initiatives in different domains, including a pilot project aimed at the sustainable production and use of locally grown agricultural products. The project also aimed to increase food self-sufficiency in New Caledonia and address a major public health issue, namely childhood and adult obesity caused by “bad food” habits.

### Lessons Learned

Limited organisational capacities and, in general, inadequate competences are the main systemic issues affecting the OCTs, implying that competence building is one of the top priorities for these territories. Consequently, bringing innovation is crucial in the development of educational and training frameworks (such as education systems, vocational training arrangements, and lifelong learning, among others) that generate and develop competences that are vital for supporting innovation and development in the various OCTs. Competence building is thus central to our systemic approach to innovation policy in these less-favoured economies^10^. However, policy learning (in both epistemic and reflexive modes) continues to be the most difficult aspect of the ongoing process of competence development and capacity building.

It also appears clear that the head of government plays a major role in all innovation-promoting actions pertaining to a specific OCT. However, this vertical coordination must be in part balanced by a form of horizontal coordination by which the central actor (i.e., the head of government) and the relevant ministries consider the actions of other stakeholders and pro-actively adjust the operating routines of the policymaking process. To improve the coordination, inclusiveness and ultimately the effectiveness of innovation policy governance, each OCT at the beginning of the TSI project has established an innovation council (or innovation advisory board). The innovation councils, which are made up of experts from the public and private sectors (e.g., government, academia, industry, unions), are expected to address several of the demands of innovation governance such as expert advice, monitoring and evaluation. As previously reported by us (Jezic von Gesseneck et al., 2018), the innovation councils play a fundamental role in the backcasting exercises that lead to the design of fine-tuned policy mixes. In each OCT, a major role is also played by the innovation director (or innovation manager), who serves as a liaison between the head of government and the innovation council (or the innovation advisory board).

In terms of policy mix design, public-private partnerships (PPPs) emerge as key policy instruments for potentially mitigating specific systemic issues. PPPs, in particular, enable OCTs to pursue non-traditional innovation paths, such as non-technological improvements, service creation, and creativity exploitation, by enlisting the participation of private actors. They also allow for the transition to open innovation, in which innovation becomes a process that spans all industries.

## Discussion and Conclusions

Developing effective innovation policies for the EU OCTs is a difficult task that requires a thorough understanding of the specific context into which the policies are implemented. Furthermore, a challenge-driven policy approach aimed at systemic innovation necessitates a long-term perspective while simultaneously exploring multiple and diverse policy pathways in the short term. Our proposed approach constructs the innovation process in such a way that the participants are supported in using specific methodologies to make their thinking more systemic (Midgley and Lindhult, 2017). As a result, the innovation process can be amplified by stakeholder dialogue to support social learning, allowing stakeholders to gain a better understanding of the possibilities and potential consequences of innovation (Colvin et al., 2014; Ison, 2016).

Concerned about daily life and the specific priorities of OCTs, we have devised a flexible approach that can be tailored to the size and characteristics of each territory. Through the lens of policy learning, we investigated how to assist three selected OCTs in overcoming organisational and institutional barriers while establishing a common orientation for innovation policies. It is worth noting that Anguilla, Curaçao, and New Caledonia all share a number of social, institutional, cultural, and historical characteristics with their respective parent countries, the United Kingdom, the Netherlands, and France. As a result, these remote territories are strongly influenced by different approaches to innovation policy and governance. Because of this unique feature, they can serve as ideal small-scale laboratories for promoting additional research at the European level.

Drawing on various literatures such as innovation studies, foresight research and political science, we offer concrete guidance to the questions of what local governments can realistically do to foster innovation processes of systemic change. Our proposed approach, in particular, may aid in the generation of critical insights for policymakers seeking to gain a distinctive competitive advantage for their highly vulnerable territories while addressing major sustainability challenges. For this reason, special consideration is also given to other key implementation policy elements such as the nature of the existing range of policy actors, the types of resources available to these actors, and the institutional arrangements under which these efforts must take place (Howlett, 2019).

A major contribution of this multiple case study is the in-depth analysis of the main systemic issues afflicting three different OCTs as well as the resulting efforts to develop effective innovation policies over time. Particular emphasis is placed on participatory policymaking, in which public and private actors actively co-create and collaborate in the identification and resolution of complex collective problems (Pierre and Peters, 2005). To enable local authorities to meet the challenge of delivering innovative solutions, a key role is played by various forms of public-private partnerships (PPPs). In this article, PPPs are primarily viewed as vehicles for promoting innovation policy (Kristensen et al., 2015) and sustainable development (Glasbergen, 2007; van Huijstee et al., 2007; Pattberg et al., 2012) in the concerned OCTs. By managing public-private interfaces, these systemic instruments are critical for these vulnerable territories to pool the best available knowledge and experience. In particular, through appropriate PPPs, it is possible to create a collaborative environment to maximise cross-disciplinary expertise among government and public knowledge organisations on the one side and private companies (especially small and medium-sized enterprises, SMEs), non-governmental organisations (NGOs) and society on the other side. According to Moreddu (2016), the main prerequisites for forming a rational partnership are shared goals, mutual benefits, and complementarity of human and financial resources. In all the concerned OCTs, the various PPPs are expected to capitalise on the respective strengths of the partners and scale-up their activities while ensuring higher-quality contributions from the private sector to innovation-oriented goals (OECD, 2016). PPPs are thus critical instruments of the innovation policy mixes required to boost both competitiveness and sustainable growth in the respective OCTs. It is important to note that our proposed approach allows governments to evaluate PPPs beyond the organisational and financial benefits of the actors involved, and assesses their contribution to the functioning of the innovation system itself. In any case, to be successful, PPPs necessitate the acquisition of new knowledge and the development of new skills by both public and private actors, particularly in relation to the achievement of the broader policy goal of system transformation.

A key question in this regard is how to shape the policy mix characteristics in order to develop and implement effective innovation policies. It is now clear that public policy outcomes, whether successful or not, are the result of dynamic processes that build and unfold over time (Compton and ‘t Hart, 2019). Therefore, we focus here on the mediating role that backcasting (as a systemic instrument) plays in policy dynamics. We argue that the proposed foresight methodology is improved by including a feedback loop that accounts for exogenous contextual changes. This results in a governance arrangement with significant advantages, given its mechanisms for monitoring, evaluation and adaptation of better strategies over time (Figure 1). This arrangement is in line with the innovation governance mode proposed by Magro and Wilson 2019, which envisions the participation of multiple stakeholders, engaged in a learning loop, to better link the strategic and policy processes.

From a dynamic and mechanistic standpoint (Capano et al., 2019), backcasting has the potential to trigger reflexive policy learning (Figure 2). This type of learning refers to knowledge utilisation that influences how policy issues are understood and perceived. In reflexive settings, where knowledge use is open-ended, outcomes are difficult to predict but exchanges are cooperative and symmetric. In this mode, uncertainty about a specific policy issue is radical and policymakers are expected to learn horizontally. This means that their attention is diffused as they interact with a diverse set of actors and perspectives. This most social type of learning occurs over time through communication, preference change, and collective puzzling (Dunlop and Radaelli, 2018b). According to Daviter (2018), reflexive learning calls into question the underlying assumptions and analytical concepts that shape policy decisions and influences how policy problems are framed for decision making (Weiss, 1979; Radaelli, 1995). As a result, reflexive learning in the policy process is fundamental to deal with the challenges of shifting problem boundaries, competing problem perceptions, and contested knowledge. In essence, reflexive learning is beneficial for stable conflict resolution and upholding legitimacy (Dunlop and Radaelli, 2019). We want to emphasise here that legitimacy is a necessary condition for the effective governance of the socio-technical and innovation systems (Borrás and Edler, 2014b). It is also worth noting that the concept of reflexivity, as the foundational component of learning-based governance (Lenoble and Maesschalck, 2010), has been debated in recent years by various transition scholars (Kemp and Loorbach, 2006; Voß and Bornemann, 2011; see also Feindt and Weiland, 2018).

By encouraging dialogue among a diverse set of actors (e.g., through backcasting), reflexive learning holds the promise of second-order learning, which is a prerequisite for systemic innovation. As a result, if the entire OCT innovation system is to be conceptualised across organisational boundaries, and sustainability values are to prevail, then reflexive learning is crucial (Quist and Tukker, 2013). This obviously is much more complex than learning at the individual level. Furthermore, those involved in social learning must gain an understanding of numerous complex feedback loops. However, we must be aware that, in times of complexity and uncertainty, acting too reflexively may lead to paralysis (Termeer et al., 2015). Indeed, if paradigmatic positions are completely incommensurable, reflexivity is dysfunctional (Pellizzoni, 2001; Dunlop and Radaelli, 2016).

Given the foregoing, it is clear that various modes of learning are required to address the systemic and complex policy issues affecting the concerned OCTs. In particular, epistemic learning is critical for reducing uncertainty and providing insights into how to understand complex phenomena (Kaplan and Berman, 2010). In our case study, epistemic policy learning is facilitated by the establishment of the innovation councils, whose members provide expert advice to relevant policy actors in a variety of technical and scientific domains. In this regard, it is important to note that the heads of the respective OCT governments lead the innovation councils and all related innovation-promoting actions. Interestingly, Charles Edquist recently highlighted a similar role for the Prime Minister within the Swedish Innovation Council (Edquist, 2019). However, one might question whether the heads of the OCT governments are capable of developing and maintaining effective leadership throughout the entire decision-making process. Our findings indicate that their leadership does not appear to be in conflict with collective, social learning. On the contrary, the leadership role of the heads of governments is a prerequisite for all multiple stakeholders to understand the value of systemic innovation and do their best to embrace and implement it in the territory for the benefit of all. The heads of governments, by leading the aforementioned innovation councils, are also critical for facilitating reflexive learning in the respective OCT contexts. Indeed, by involving local epistemic communities in backcasting exercises, these advisory bodies promote social debate and deliberation. Local experts are thus removed from closed dyadic relationships with policymakers and placed in much larger social networks, enhancing the policy capacity of the investigated OCTs. The ability to combine reflexive and epistemic learning modes in a structured manner is undoubtedly the most innovative aspect of the proposed methodology aimed at implementing appropriate innovation strategies and policies in these remote territories.

In conclusion, we argue that, in order to align with evolving strategies, appropriate policy mixes should be developed and better shaped over time through a collective, social endeavour. As a result, the main innovation challenge for OCTs and other remote territories is to re-think policymaking in a more radical and transformative way. This necessitates a new common approach to addressing their systemic and complex issues, which facilitates policy learning and involves more actors in the policy process. In terms of innovation policy and governance, this foresight approach could be used to promote sustainable development and transformation in other less-favoured territories, such as SIDS and underperforming EU regions. For example, it might be interesting to investigate how backasting could be used in specific EU territories to implement more cohesive innovation strategies in order to accelerate Europe's recovery.

## Data Availability Statement

The raw data supporting the conclusions of this article will be made available by the authors, without undue reservation.

## Author Contributions

MJ conceived the broad idea while RT developed the foresight-based methodology. RT wrote the initial manuscript. MJ extensively revised the paper. MJ and RT finalised and approved for publication. All authors contributed to the article and approved the submitted version.

## Funding

This work was supported in part by the Italian Ministry of University and Research through 5 × 1,000 2020 (ARCHES, Castellana Grotte, Bari).

## Conflict of Interest

The authors declare that the research was conducted in the absence of any commercial or financial relationships that could be construed as a potential conflict of interest.

## Publisher's Note

All claims expressed in this article are solely those of the authors and do not necessarily represent those of their affiliated organizations, or those of the publisher, the editors and the reviewers. Any product that may be evaluated in this article, or claim that may be made by its manufacturer, is not guaranteed or endorsed by the publisher.

## Footnotes

^1^As a result of Brexit, the number of Overseas Countries and Territories associated with the EU has been reduced from 25 to 13.

^2^Other terms have been used to describe similar situations. These include persistent problems (Rotmans and Loorbach, 2009) or wicked problems (Rittel and Webber, 1973; Blok et al., 2016).

^3^As suggested by Rogge and Reichardt (2016), we distinguish between instrument mix and policy mix, with the latter encompassing the former. Specifically, we incorporate policy strategy as one of the elements in the policy mix concept.

^4^Backcasting can be defined as imagining a desirable future (e.g., a vision) and then returning to the present to plan how to achieve it (Quist, 2007). It has gradually gained popularity and widespread use over the years. This is most likely due to the growing acceptance of the normative concept of sustainability (Renn et al., 2009; Wang, 2011).

^5^See Jezic von Gesseneck et al. (2018) for a description of the initial foresight methodology.

^6^Isla, the refinery run by neighbouring Venezuela's state oil company PDVSA, is the second-biggest in the Caribbean but has long been plagued by technical problems.

^7^The term epistemic community has been defined in a variety of ways. Here we adopt the widely accepted definition provided by Haas (1992, p. 3): “*An epistemic community is a network of professionals with recognised expertise and competence in a particular domain and an authoritative claim to policy relevant knowledge within that domain or issue-area”*.

^8^There is no widely recognised definition of PPP. Here we adopt the general definition from the Ministry of Foreign Affairs of the Netherlands (2010): “*A form of cooperation between government and business (in many cases also involving NGOs, trade unions and/or knowledge institutions) in which they agree to work together to reach a common goal or carry out a specific task, jointly assuming the risks and responsibility and sharing their resources and competences”*.

^9^Strong divisions and inequalities continue to exist between the native Kanak people and the Europeans.

^10^We propose three-pillar blended learning (see Table 1) as a very flexible approach for competence building and lifelong learning. Three pillar-blended learning is a combination of face-to-face learning, learning-by-doing and learning online.
